# The Influence of Ethnicity on the Development of Type 2 Diabetes Mellitus in Women with Gestational Diabetes: A Prospective Study and Review of the Literature

**DOI:** 10.5402/2012/341638

**Published:** 2012-04-17

**Authors:** Christian M. Girgis, Jenny E. Gunton, N. Wah Cheung

**Affiliations:** ^1^Diabetes and Transcription Factors Group, Garvan Institute of Medical Research, Darlinghurst, Sydney, NSW 2010, Australia; ^2^Faculty of Medicine, University of Sydney, Sydney, NSW 2006, Australia; ^3^Department of Diabetes and Endocrinology, Westmead Hospital, Weatmead, NSW 2145, Australia; ^4^St Vincent's Clinical School, University of NSW, Sydney, NSW 2010, Australia

## Abstract

As the worldwide prevalence of type 2 diabetes continues to rise at an alarming rate, the search for susceptible populations likely to benefit from preventative measures becomes more important. One such population is women with a previous history of gestational diabetes mellitus (GDM). In this prospective study of 101 women who had GDM in Australia, ethnicity was a major risk factor for the development of diabetes following a diagnosis of GDM. With a mean followup of 5.5 years after GDM, South Asian women had a significantly higher risk of developing abnormal glucose tolerance (AGT) (69%) than women of all other ethnicities (*P* < 0.05). The prevalence of diabetes and impaired glucose tolerance was also very high amongst other groups: South East and East Asian (11/27, 41%), Middle-Eastern (8/18, 44%), South European backgrounds (5/12, 42%), and Australian-born women 39% (11/28). A review of the literature supports the role of ethnicity in the development of diabetes amongst these women. These findings have implications for South Asian countries and countries such as Australia where there is a population from diverse ethnic backgrounds and where the implementation of targeted measures to stem the growing tide of diabetes is needed.

## 1. Introduction

The prevalence of type 2 diabetes is rising at an alarming rate. A recent report by the International Diabetes Foundation projected that, by 2030, up to 438 million people may be affected by this disease, accounting for more than 4.5% of the world's projected population [1]. In China alone, a recent survey reported that 92.4 million adults (approximately 9.7% of the nation's population) have diabetes [2] while another report estimated that up to 19.4 million individuals living in the Indian subcontinent were affected [3]. In another “hot spot,” namely, the Middle East, the prevalence of diabetes ranges from 13 to 19% amongst 20 to 79 years old in several countries [4]. The West has not been spared. Studies from Australia show that the prevalence of diabetes has doubled from 3.4% in 1981 [5] to 7.4% in 1999-2000 [6].

In the light of these alarming global trends, the search for effective preventative measures to target individuals at greatest risk of diabetes is gaining momentum. Women with a prior history of gestational diabetes mellitus (GDM) represent one such susceptible population. In fact, the original criteria for GDM, as defined in 1964, were based on the likelihood of the mother developing diabetes in later life [7].

GDM affects 1.4% to 12.3% of pregnancies depending on the populations tested and the diagnostic criteria used [8]. Recent evidence also suggested that the prevalence of GDM *itself* is on the rise, possibly in parallel with the increasing prevalence of type 2 diabetes. In the Kaiser Permanente of Colorado Screening Program, the incidence of GDM doubled from 2.1 to 4.1% amongst 36,403 pregnancies from 1994 to 2002 [9]. Women of various ethnicities including those from Asian, African American, and Hispanic backgrounds were twice as likely to experience GDM in this study as compared to those of non-Hispanic White extraction. Another study from Australia reported a significantly higher incidence of GDM amongst women who had migrated from Asian, Middle Eastern, and Mediterranean countries (7.2–15%) as compared to those born in Australia or New Zealand (4.3%) [10]. Amongst women attending the antenatal clinic at our centre from 1988 to 1996, we also reported a high incidence of GDM amongst migrant women of Chinese (9.5%), Filipino (6.7%), Sri Lankan (10.5%), and Vietnamese backgrounds (9.7%) [11]. Therefore, by virtue of their greater risk of GDM, women from certain ethnicities may be more susceptible to developing diabetes in the long term.

It is also possible that ethnicity *per se* may influence the subsequent development of diabetes amongst women with previous GDM. In an indirect comparison of two similarly designed studies, 62% of women with a history of GDM from Trinidad were subsequently diagnosed with type 2 diabetes [12] whereas only 3.4% of Swedish women in another study developed the disease (followup 3–6 yrs) [13]. However, there are relatively few studies directly examining differences in the prevalence of diabetes amongst women with previous GDM pregnancies on the basis of their ethnicity.

Potential ethnic differences in the development of disease are also relevant to countries such as Australia where massive immigration since 1945 has led to the influx of nearly 7 million people, a substantial proportion of whom originate from Asian countries and the region of Oceania (http://www.immi.gov.au/media/fact-sheets/02key.htm). The demonstration of a particularly high incidence of type 2 diabetes following an episode of GDM amongst certain ethnic groups may assist in targeting at-risk individuals for screening and preventative strategies, thereby potentially lessening the burden of type 2 diabetes in these communities.

To address this issue, we studied two groups of women with a previous history of GDM, namely, migrant women of non-Anglo-Celtic background and women born in Australia. Our aim was to identify differences in the long-term incidence of impaired glucose tolerance (IGT) or diabetes between these groups of women that could potentially influence the public health policy approach towards the growing tide of diabetes in our country, Asia, the Indian subcontinent, and the Middle East.

## 2. Materials and Methods

Study participants were recruited from two centres, namely, Westmead and Nepean Hospitals, located in western Sydney, Australia. The catchment area is a demographically diverse region characterized by a high proportion of foreign-born residents (21% of the population). The participants were previously diagnosed with GDM and had attended the antenatal clinics at one of these hospitals between 1988 and 1994. Women who had migrated to Australia and were not from Anglo-Celtic backgrounds were systematically recruited for study inclusion. These women were further grouped according to their country of birth under the regions of South Asia (India, Sri Lanka, and Fijian Indians), Southeast and East Asia (China, Korea, Singapore, Vietnam, Laos, Philippines, Malaysia), Southern Europe (Italy, Malta, former Yugoslavia, Greece), or the Middle East (Egypt, Syria, Iran, Iraq, Lebanon). A group of women born in Australia was randomly selected for comparison.

The study protocol was similar to that previously reported by our centre [14]. In brief, medical records of women with GDM were reviewed and data including the country of birth, the results of the pregnancy GTT, initial weight and height, age at the time of conception, family history, and obstetric history were recorded. Subjects were then contacted by phone and asked if they had developed diabetes, and if not, if they were willing to be tested, by GTT. Where the subject declared that she already had diabetes, verification was made with her local doctor.

Statistics were performed in SPSS version 14, (Chicago, IL, USA). Results are presented as mean ± SD. Correlation between variables was performed by logistic regression analysis. Comparison of subjects with nonparticipants was analysed by student's *t*-test. *P* values less than 0.05 were considered significant.

## 3. Results

We identified 352 women with GDM from the above-migrant backgrounds. Of these, 191 were not contactable. Of the remaining 161 subjects, 88 were not interested in participating in the study. Therefore, 73 participants remained. A randomly selected group of 28 Australian-born women (age 34 ± 4.6 yrs) with a previous history of GDM were included for comparison.

The mean duration since the subjects' first GDM pregnancy, to the time of testing or the development of AGT, was 5.5 years (range 1–12). Of the 73 overseas born women, 24 (33%) had already developed diabetes or were found to have diabetes upon testing. A further 11 (15%) had IGT. Thus in total, 48% of these women had either diabetes or IGT (collectively termed Abnormal Glucose Tolerance, AGT). Of those with AGT, 17 (49%) were newly diagnosed by recall for this study (41% with diabetes, 59% IGT).

**Figure 1:**
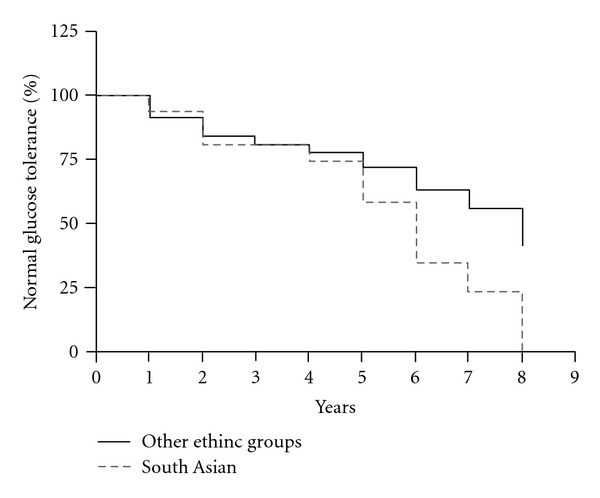
Survival analysis showing the increased progression to AGT amongst subjects of South Asian extraction versus other women, *P* < 0.05.

 The mean age at the time of diagnosis of AGT was 35.5 ± 5.4 years. When examined by region of birth, South Asians had the highest rate of AGT at 69%, a significantly higher proportion than the other ethnic groups combined (*P* = 0.039, Cox-Mantel survival, Figure 1). However, all groups had a high rate of post-GDM diabetes or impaired glucose tolerance. Women of Middle-Eastern origin had a 44% risk of AGT, South Europeans 42%, South East Asians 41%, and Australian-born women 39%.

Further analysis was performed to examine the relationship of pregnancy factors with the development of AGT amongst the immigrant women (Table 1). Women who had developed AGT were 3.9 times more likely to have had pregnancy-induced hypertension (PIH) during their index pregnancy, 2.8 times more likely to have required insulin during that pregnancy, and had higher glucose results at each time-point during the pregnancy glucose tolerance test. Women with a subsequent normal (non-GDM) pregnancy had an odds ratio for developing AGT of 0.2 compared to women without subsequent normal pregnancy.

On multiple logistic regression analysis of all the significant factors listed in Table 1, the GTT result at 2 hours (OR 2.5, 95% CI 1.4–4.5, *P* = 0.002) and the number of episodes of GDM (OR 4.0, 95% CI 1.4–11.3, *P* = 0.008) were independent predictors for the development of AGT.

There were also significant differences in anthropometric variables amongst the ethnic groups (Table 2). South Asians and South East Asians with AGT were significantly lighter and had lower BMI than those of Middle-Eastern or South European extraction who had developed AGT (*P* < 0.05 all). Similarly, South East Asians and Caucasians with NGT were lighter than their Middle-Eastern and South European counterparts with NGT (*P* < 0.05). Height was not found to be associated with the development of AGT in the different ethnic groups.

## 4. Discussion

The risk of diabetes following an episode of GDM is substantially higher than the baseline risk of diabetes. A recent meta-analysis of 6 controlled followup studies determined that the risk of developing diabetes after GDM was 6 times greater than in parous women who had not suffered GDM (RR 6.0, 95% CI 4.1–8.8) [15]. Furthermore, a considerable proportion of parous women with diabetes, that is, 10–31%, would have previously experienced an episode of GDM prior to the development of frank diabetes [15]. A possible reason for this is that GDM represents an underlying state of limited beta-cell reserve that becomes clinically manifest only at a time of marked insulin resistance, namely, late pregnancy [16]. Therefore, to target this distinct subset of susceptible women with screening and preventative measures may potentially lessen the burden of diabetes and have significant public health implications.

Women with previous GDM represent a substantial proportion of the population that may be difficult to target as a whole. The search for additional factors that would indicate an even greater risk of diabetes amongst women with GDM may further assist in targeting prevention. Such risk factors include the use of insulin in pregnancy, high BMI, recurrent GDM, higher glucose levels in oral glucose tolerance testing, and fasting hyperglycaemia during pregnancy [14, 17]. Ethnicity appears to be a major risk factor. In fact, a review of the literature (Table 3) indicates that consistent with these results, women with GDM of Asian background in Western countries are more likely to subsequently develop diabetes that women of Anglo-Celtic origin [17–20].

A recent large cohort study of 5470 GDM patients and 783 control subjects reported a significantly higher incidence of diabetes amongst Asian women who had suffered GDM as compared to Caucasian controls over a 9-year period (HR 2.1, 95% CI 1.7–2.7) [17]. In earlier analysis of this cohort, the two-fold increase in the prevalence of diabetes amongst Asian versus non-Asian women had become apparent at just 6 months postpartum [18].

Another Australian study found that Vietnamese-born mothers were significantly more likely to develop GDM than their Australian-born counterparts (7.8% versus 4.3%) [19]. Furthermore, the incidence of diabetes within 9 years after GDM was substantially higher amongst women born in Vietnam (25%, 17/68) compared to Australian-born women (9%, 52/581) [19].

Amongst 221 women in a UK study, 35% of Indo-Asian subjects had persistent glucose intolerance 3 months postpartum compared with 7% of Caucasian and 5% of Afro-Caribbean subjects [20].

In other countries, Latino women and various indigenous populations have also been identified to be at high risk [21, 22].

As well as a greater incidence of diabetes, women of non-English speaking background also appear to develop diabetes* within a shorter time* following their episode of GDM than their Anglo-Celtic counterparts [23].

The current study further supports the influence of ethnicity on the incidence of GDM and the subsequent development of diabetes. When tested 1–12 years later, 48% of women of non-Anglo-Celtic migrant backgrounds had developed abnormal glucose tolerance. Thirty three percent of patients had diabetes, and 15% had IGT. This was in comparison to 39% of Australian-born women who had developed abnormal glucose tolerance at the time of followup. Women from South Asian countries were even more likely to develop diabetes than the other groups. By 8 years, South Asians displayed a significantly higher prevalence of AGT than the other women. On the whole, the prevalence of diabetes amongst these women was substantially higher than that of the general Australian population at the time of the study, estimated to be in the order of 4–6% in that age group [24]. It is also likely that with longer duration of followup, a greater proportion of study participants would have developed diabetes. In fact, one well-cited study reported a rate of subsequent diabetes of 49.9% over a followup of 22–28 years in an American population with previous GDM [25].

There were significant differences in anthropometric parameters, specifically weight and body mass index, amongst those from different ethnic groups with AGT (Table 2). South Asians, South East Asians, and Australian-born women with AGT were significantly lighter and had lower BMIs than their Middle-Eastern and South European counterparts with AGT (*P* < 0.05 for all), suggesting that simple obesity is not the only predictor of glucose intolerance. The clinical heterogeneity of those at risk of diabetes is well established and recent guidelines have recommended the use of alternate BMI cutoffs in the definition of overweight and obesity amongst particular ethnic populations, particularly those of Asian extraction [26].

There are limitations to this study. Firstly, as is usually seen in followup after GDM studies, there was a high rate of nonparticipation. Women who participated were more likely to have a positive family history than those who declined to participate (44% versus 24%, *P* = 0.04). It is also possible that participants with a known abnormal glucose tolerance were more likely to volunteer information about their status. Women from South-East Asia were more likely to decline to participate (38% of participants were from SE Asia versus 61% of nonparticipants, *P* = 0.02). Another limitation was the lack of systematic recruitment of Australian-born women, who were a randomly selected group included for comparison. Although born in Australia, some of these women were not of Anglo-Celtic extraction, which would tend to minimise differences. However, the numbers were sufficient to demonstrate a significantly greater prevalence of diabetes amongst those from South Asian countries compared to the combination of women from all other groups, and a very high rate of AGT overall.

 What are some of the potential mechanisms by which ethnicity might influence the subsequent development of type 2 diabetes in those with GDM?

Firstly, genetic factors are likely to play an important role. Genome-wide association studies of 283 Danish women who had suffered GDM compared to 2446 glucose-tolerant controls identified a number of genetic loci associated with GDM [27]. The similarity of these loci to those associated with type 2 diabetes, namely, TCF7L2 and CDKAL1, led the authors to propose that GDM and type 2 diabetes were different manifestations of the same genetic entity. However, the potential impact of particular genetic variants on the subsequent development of type 2 diabetes in women with GDM is yet to be determined but merits consideration.

Possible differences in the long-term pathophysiological response to the heightened insulin resistance of pregnancy may also play a role. A recent study examining 60 hispanic women with recent GDM who were followed over 52 months reported that the effect of weight gain on the subsequent risk of type 2 diabetes was secondary to changes in serum adiponectin levels and the degree of insulin resistance (as quantified by the disposition index) [28].

The low participation rate amongst women of Southeast and East Asian origin possibly reflects a reduced awareness of the risks of GDM and a general lack of information regarding healthy living amongst this population. Language barriers amongst migrants may make the promotion of better health amongst this population difficult [29]. There are also specific cultural and social barriers to healthy lifestyle that have been identified in migrant women who have had GDM [30]. Migrants are also more likely to be socially disadvantaged and have lower income than those born locally, with potential impacts on their overall health [31]. Furthermore, dietary and lifestyle behaviour is largely influenced by an individual's cultural background and these factors may subsequently influence the risk of diabetes. In support of this, a study of Korean women with GDM who consumed a higher intake of fat calories compared to total calories in the postpartum period was more likely to develop impaired glucose tolerance and diabetes [32]. In addition, the change in lifestyle amongst those migrating to Western societies characterized by high-calorie diets and sedentary lifestyles may play a role, particularly where the underlying susceptibility to diabetes is high [33].

In public health terms, the high incidence of diabetes after GDM has the potential to be a major problem for a country like Australia where 20% of the population is foreign born [27]. The relatively young age at which migrant women with GDM developed AGT (i.e., 35.5 years) is also cause for major concern due to the long-term threat posed by diabetes, its inherent complications, and cost to the health care economy. Data from the 1990s in US suggest that a reduction of type 2 diabetes by 25% over 10 years, amongst women who have had GDM, would save the country $179 million [34]. Conversely, failure to address this issue will result in a significant human and financial cost in the years ahead.

Although prevention is better than cure, the search for cost-effective and pragmatic preventative measures targeted at the right population of women with GDM is a challenge. To date, there have been only two drug trials to specifically intervene in women with GDM or a history of GDM to prevent diabetes. In one drug trial to specifically intervene in women a history of GDM to prevent diabetes, the use of troglitazone was subsequently discontinued because of reports of drug-induced hepatotoxicity in other populations [35]. Data from other at-risk groups show that diet and exercise may reduce the incidence of type 2 diabetes. A Chinese study demonstrated that intensive dietary or exercise management over 6 years reduced the likelihood of patients with IGT developing diabetes by about 40% [36]. Similarly, amongst males in Japan with IGT, there was a significant 67% reduction in the incidence of diabetes amongst those who received dietary counseling and participated in exercise over 4 years as compared to controls [37]. Another study found that women who exercised at least once a week were 30% less likely to develop diabetes than those who were sedentary [38]. One would predict that such measures might be similarly effective in preventing type 2 diabetes in women who have had GDM. In the Diabetes Prevention Program, 16% of the women with impaired glucose tolerance enrolled in the study had a past history of GDM. Subgroup analysis suggested that both lifestyle intervention and metformin were effective in preventing progression to diabetes in this cohort [39].

## 5. Conclusion

We have found that there is a very high prevalence of abnormal glucose tolerance amongst women living in Sydney, Australia who have had GDM. This presents public health policymakers a unique opportunity to counter the growing threat of diabetes by aiming preventative measures at this high-risk group. However, the challenges faced by such policies need to be better defined, specifically the relative effects of socioeconomic and financial inequality, language, and cultural barriers in the progression of migrant women from GDM to diabetes. Research examining the preventative value of exercise and lifestyle modification in the prevention of diabetes amongst women with GDM is also needed to guide the way forward.

## Figures and Tables

**Table 1:** Risk factors for the development of abnormal glucose tolerance following gestational diabetes.

	Abnormal glucose tolerance, *N* = 35	Normal glucose tolerance, *N* = 38	Odds ratio (95% CI)	*P* value
Family history of DM	51%	39%	1.6 (0.6–4.1)	NS
PIH in IP	31%	8%	5.3 (1.3–21.2)	**0.02**
Macrosomia in IP	20%	24%	0.8 (0.3–2.5)	NS
Insulin required in IP	51%	18%	4.5 (1.6–13.1)	**0.005**
Age at IP	30.8 ± 4.7	31.7 ± 4.8	1.0 (0.9–1.1)	NS
BMI in IP	27.1 ± 6.1	25.7 ± 6.4	1.0 (1.0–1.1)	NS
Fasting BSL in IP	5.6 ± 1.5	4.8 ± 0.7	2.8 (1.2–6.6)	**0.02**
1-hour BSL in IP	11.5 ± 2.6	10.3 ± 1.2	1.6 (1.0–2.4)	**0.04**
2-hour BSL in IP	10.3 ± 2.8	8.3 ± 1.4	2.1 (1.3–3.3)	**0.003**
GDM episodes	1.6 ± 0.7	1.3 ± 0.6	2.2 (1.0–4.8)	**0.04**
Subsequent non-GDM pregnancies	0.1 ± 0.3	0.5 ± 0.7	0.2 (0.06–0.6)	**0.006**
Years after IP	6.7 ± 2.7	6.0 ± 2.1	1.1 (0.9–1.4)	NS
Age at followup	37.5 ± 5.4	37.6 ± 5.1	1.0 (0.9–1.1)	NS

PIH = pregnancy induced hypertension. IP = index pregnancy.

**Table 2:** Anthropometric and other characteristics of study participants.

	South Asian (*n* = 16)			South East Asian (*n* = 27)			Middle-Eastern + South European (*n* = 30)			Australian-born (*n* = 28)		
	NGT	AGT	*P*	NGT	AGT	*P*	NGT	AGT	*P*	NGT	AGT	*P*
Age (yrs)	32.6 ± 3.5	31.4 ± 1.6	0.76	31.2 ± 1.0	30.6 ± 1.6	0.54	31.4 ± 1.1	30.6 ± 1.1	0.63	31.6 ± 1.3	31.9 ± 1.4	0.80
Weight (kg)	58.2 ± 3.2	62.2 ± 4.2	0.46	52.7 ± 3.3	60.0 ± 5.5	0.28	75.8 ± 3.4	83.8 ± 4.7	0.18	72.1 ± 3.7	68.2 ± 4.5	0.84
BMI (kg/m^2^)	24.4 ± 1.2	25.4 ± 1.2	0.59	22.0 ± 1.6	24.4 ± 1.8	0.32	29.7 ± 1.4	33 ± 1.8	0.15	29.7 ± 1.4	33 ± 1.8	0.61
GTT 0 hr (mmol/L)	5.0 ± 0.2	5.1 ± 0.2	0.55	4.5 ± 0.1	5.5 ± 0.7	0.18	5.1 ± 0.2	6.3 ± 0.5	**0.04**	5.1 ± 0.2	6.3 ± 0.5	0.96
GTT 2 hr (mmol/L)	8.5 ± 1.0	10.1 ± 0.5	0.21	8.5 ± 0.4	10.2 ± 1.3	0.21	7.9 ± 0.3	10.5 ± 0.9	**0.03**	7.1 ± 0.3	10 ± 1.0	0.99
Parity	1.8 ± 1	0.6 ± 0.2	0.34	1.2 ± 0.4	0.7 ± 0.3	0.36	2.7 ± 0.5	2.1 ± 0.8	0.81	0.88 ± 0.3	1.1 ± 0.3	0.22

**Table 3:** Studies comparing the incidence of diabetes amongst Asian versus Anglo-Caucasian with a history of GDM.

	Number of participants with GDM	Followup	Findings
[17]	5470	9 years	Higher incidence of T2DM amongst Asians versus Caucasians (HR 2.1, 95% CI 1.7–2.7)
[18]	2,957	6 months	Higher incidence of T2DM amongst Asians (3.5%) versus North Europeans (1.2%)
[19]	649	9 years	Higher incidence of T2DM amongst Vietnamese-born (25%) versus Australian-born women (9%)
[20]	221	3 months	Higher incidence of IGT amongst Indo-Asians (35%) versus Caucasians (7%) or Afro-Caribbean (5%)
